# Laparoscopic reduction of intersigmoid hernia: early surgical intervention for a rare form of internal hernia

**DOI:** 10.1093/jscr/rjac003

**Published:** 2022-02-15

**Authors:** Sang Il Youn, Dong-Wook Kim, Ye Seob Jee

**Affiliations:** Department of Surgery, Dankook University College of Medicine, Cheonan, Republic of Korea; Department of Surgery, Dankook University College of Medicine, Cheonan, Republic of Korea; Department of Surgery, Dankook University College of Medicine, Cheonan, Republic of Korea

## Abstract

Intersigmoid hernia is a rare form of internal hernia presenting with symptoms of bowel obstruction. A 32-year-old male visited the emergency department with chief complaint of abrupt onset of abdominal pain without any history of prior abdominal surgery. The initial abdominal X-ray and computed tomography (CT) scan exhibited mild distension of small bowel and paralytic ileus with no definitive obstruction site. However, a 12-h follow-up abdominal X-ray showed manifestations of newly appeared step-ladder sign and the CT scan displayed mechanical obstruction in the left lower quadrant area. Upon laparoscopic examination, herniation of small bowel was observed through the intersigmoid recess. Reduction was performed for about 5 cm of incarcerated ileum, and there was no sign of necrosis or lasting damage. The patient was discharged without complications. Laparoscopic management of intersigmoid hernia is possible with early surgical management of mechanical obstruction.

## INTRODUCTION

Intersigmoid hernia is a rare form of internal hernia, defined as herniation of the small intestine into the intersigmoid fossa through the intersigmoid rescess. The entrapment of small bowel through this defect often presents with symptoms of obstruction. Owing to the rare accounts of incidence and difficulty of diagnosis before surgery, intersigmoid hernia may often be overlooked for diagnosis. Since mortality is reported up to 50% with strangulation [1], early impression and prompt surgical intervention of internal hernia is crucial for successful management. Through this report we present a case of intersigmoid hernia treated by laparoscopy.

## CASE REPORT

A 32-year-old male visited the emergency department with chief complaint of abrupt onset of abdominal pain. The patient had a past medical history of receiving thoracic surgery for patent ventricular septal defect 30 years ago but had no history of prior abdominal surgery. The patient’s vital signs were stable with no fever. Upon physical examination, the patient showed non-distended soft abdomen with mild tenderness in the left lower quadrant (LLQ). There were no signs of rebound tenderness or muscle guarding nor any other evidence indicating peritonitis. The abdominal X-ray and computed tomography (CT) scan exhibited mild distension of small bowel and paralytic ileus with no definitive obstruction site. The laboratory data showed elevated peripheral white blood cell (WBC) count of 12 030/mm3 and normal C-reactive protein level of 0.06 mg/dl.

**Figure 1:**
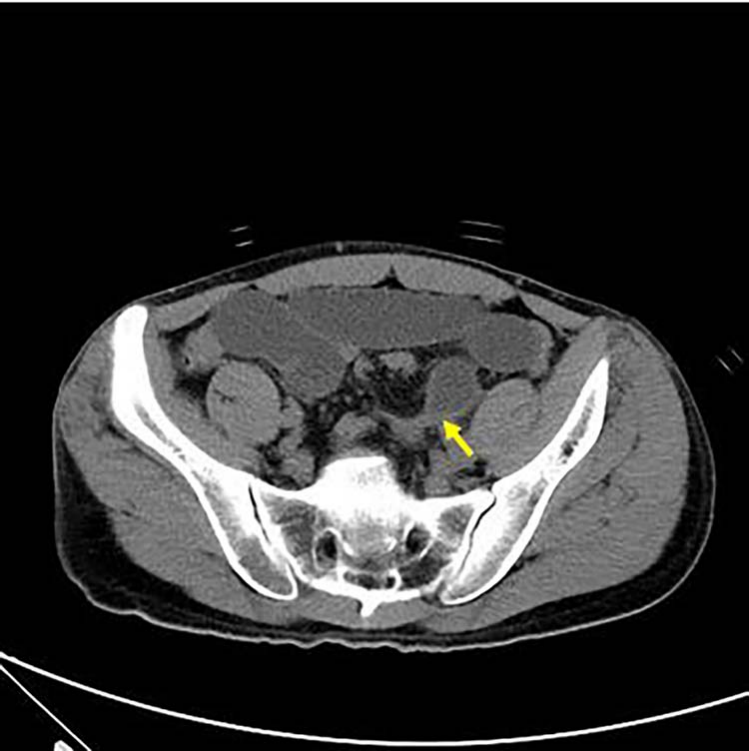
Axial view of abdominal CT revealing intersigmoid hernia with arrow indicating the leading point.

**Figure 2:**
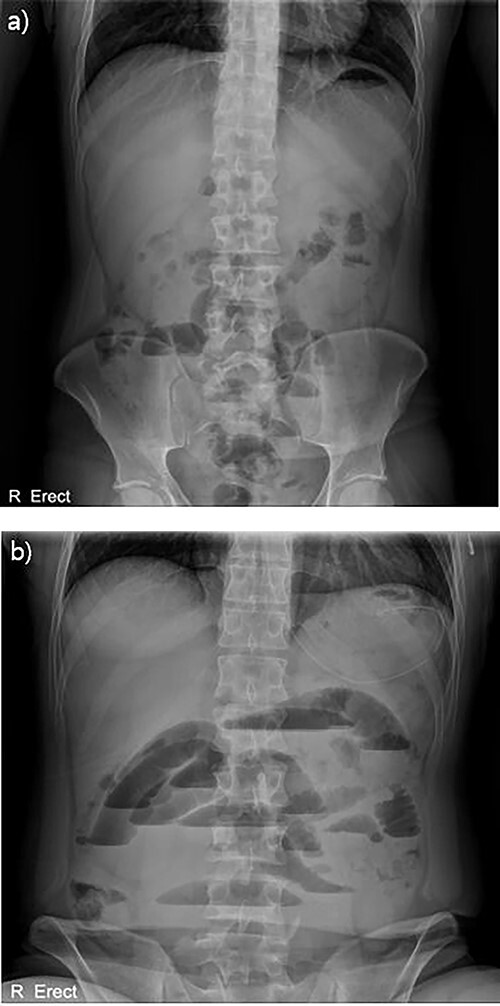
Abdominal X-ray findings (**a**) initial findings before admission; (**b**) 12-h follow-up findings revealing step ladder sign.

With no indications for immediate surgical intervention, the initial treatment plan for the patient was decided for conservative management with non per os (NPO) and Levin tube drainage. The patient, however, complained of aggravated abdominal pain after 12 h of conservative care, with elevated WBC count of 15 590/mm3. Changes were found in the abdominal X-ray showing manifestations of newly appeared step-ladder sign (Fig. 1a and b), and the CT scan displayed aggravated dilation of small bowel with a leading point of the mechanical obstruction in the LLQ area (Fig. 2).

**Figure 3:**
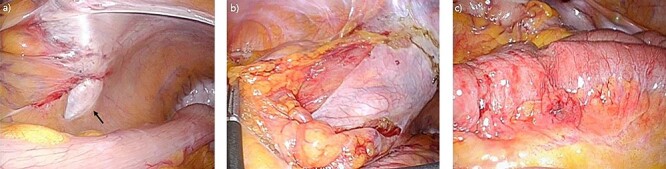
Laparoscopic findings (**a**) arrow indicating hernia orifice of the intersigmoid rescess; (**b**) wide opened hernia orifice; (**c**) laparoscopic finding of incarcerated segment of ileum.

The patient was immediately scheduled for emergency operation. Laparoscopy was performed with 12-mm port for the three-dimensional scope in the umbilicus and two 5-mm ports were inserted into the right lower quadrant and suprapubic area for the working port. Upon examination, herniation of small bowel was observed through a defect between the sigmoid colon and the parietal peritoneum (Fig. 3a). Reduction of about 5 cm of incarcerated ileum was carried out with relative ease using bowel forceps. Erythema was noted in the released ileum, but there was no sign of necrosis or lasting damage (Fig. 3c). Thereafter, the hernia defect between the mesosigmoid and the parietal peritoneum was wide opened to prevent recurrence (Fig. 3b). The small bowel was double checked from the Treitz ligament to the ileocecal valve for examination of possible other lesions and the surgery was completed. The patient was discharged on the seventh postoperative day without immediate complications. A follow-up appointment after discharge revealed that the patient was in good condition without any symptoms.

## DISCUSSION

Internal hernia is a rare disease entity, which is reported to account for 0.2 to 6% of small bowel obstruction [2–4]. A large portion of internal hernia is attributable to postoperative causes such as Petersen’s hernia or herniation through mesenteric defect created from reconstruction after gastrectomy or small bowel anastomosis [5]. Small bowel obstruction occurring after such surgery can be managed promptly and accordingly by clear impression, but if there is no surgical history, multiple diseases including malignancy should be considered based on patient age and past medical history. Out of many differential diagnoses, mesosigmoid hernia should be suspected when there is a leading point on the LLQ side.

Hernias involving the mesosigmoid were classified into three types by Benson and Killen [6]: intersigmoid, transmesosigmoid and intramesosigmoid types. It is reported that only about 6% of internal hernia is attributable to hernias in the mesosigmoid [1]. Since the incidence is extremely rare, caution should be paid to the part that diagnosis could be delayed.

Nowadays, in the era of minimally invasive surgery, laparoscopy might be the initial choice for the surgery of small bowel obstruction. Previous cases on intersigmoid hernia report successful implementation of laparoscopy, which can satisfy both the therapeutic purpose and the diagnostic purpose simultaneously [7–9]. Decision can be made intraoperatively, depending on the feasibility, whether the operation may be done by laparoscopy or an open conversion may be necessary. The opening of the hernia may be dissected and open wide or closed by suturing [9]. There is no consensus as to which method is superior, but we believe either method will suffice. In our case, the angle of the laparoscopic working arm and the extent of bowel distension were not plausible for endosuturing, and therefore we chose to open up the hernia orifice to avoid possible injury to the underlying iliac vessel and ureter.

In order for the treatment to end simply by laparoscopy, prompt diagnosis and surgical intervention are essential. In our case, even though the duration of conservative management was only 12 h, there was some degree of disturbance in the visual field due to the distention of the small bowel. Had there been no impression of leading point in the LLQ area, there was a high possibility that conversion to laparotomy should have been necessary. In addition, despite soft and careful manipulation of the intestine, serosa injury was inevitable to the edematous and distended bowel, which required seromuscular repair.

We hereby report a case of intersigmoid hernia which was successfully managed by laparoscopy. Prompt diagnosis and early surgical intervention is essential to prevent strangulation and for the treatment to be completed by laparoscopy.
